# Vaccination before or after SARS-CoV-2 infection leads to robust humoral response and antibodies that effectively neutralize variants

**DOI:** 10.1126/sciimmunol.abn8014

**Published:** 2022-01-25

**Authors:** Timothy A. Bates, Savannah K. McBride, Hans C. Leier, Gaelen Guzman, Zoe L. Lyski, Devin Schoen, Bradie Winders, Joon-Yong Lee, David Xthona Lee, William B. Messer, Marcel E. Curlin, Fikadu G. Tafesse

**Affiliations:** ^1^ Department of Molecular Microbiology & Immunology, Oregon Health & Science University; Portland, OR 97239, United States.; ^2^ Division of Infectious Diseases, Oregon Health & Science University; Portland, OR 97239, United States.; ^3^ Biological Sciences Division, Pacific Northwest National Laboratory, Richland, WA 99354, United States.; ^4^ OHSU-PSU School of Public Health, Oregon Health & Science University; Portland, OR 97239, United States

## Abstract

Current COVID-19 vaccines significantly reduce overall morbidity and mortality and are vitally important to controlling the pandemic. Individuals who previously recovered from COVID-19 have enhanced immune responses after vaccination (hybrid immunity) compared to their naïve-vaccinated peers; however, the effects of post-vaccination breakthrough infections on humoral immune response remain to be determined. Here, we measure neutralizing antibody responses from 104 vaccinated individuals, including those with breakthrough infections, hybrid immunity, and no infection history. We find that human immune sera following breakthrough infection and vaccination following natural infection, broadly neutralize SARS-CoV-2 variants to a similar degree. While age negatively correlates with antibody response after vaccination alone, no correlation with age was found in breakthrough or hybrid immune groups. Together, our data suggest that the additional antigen exposure from natural infection substantially boosts the quantity, quality, and breadth of humoral immune response regardless of whether it occurs before or after vaccination.

## Teaser

SARS-CoV-2 infection combined with vaccination improves antibody quality similarly regardless of the order of these events.

## INTRODUCTION

Severe acute respiratory coronavirus 2 (SARS-CoV-2) is the causative agent of the ongoing coronavirus disease 2019 (COVID-19) pandemic. Globally, cases continue to increase despite worldwide vaccination campaigns. (*1*) Numerous safe and effective vaccines have been developed which effectively reduce the risk of infection, severe disease, and death including BNT162b2 (Pfizer), mRNA-1273 (Moderna), and Ad26.COV2.S (Janssen). (*2*, *3*) However, variants of concern (VOC) with differing levels of increased transmissibility and resistance to existing immunity have sequentially emerged, spread widely and receded over time since the beginning of the pandemic. (*4*–*7*) Several studies have shown that antibody responses from the initial wave of vaccines in early 2021 have waned over the six months following vaccination, possibly contributing to an increase in breakthrough infections. (*8*–*12*) Booster vaccine doses were first approved in Israel in July 2021, and have since been more widely adopted in other countries to address these concerns despite the concern that boosters campaigns may divert much needed vaccine doses away from lower income countries. (*13*)

Vaccination following recovery from natural SARS-CoV-2 infection, or “hybrid immunity,” has been reported to substantially increase both the potency and breadth of humoral response to SARS-CoV-2. (*14*, *15*) However, current studies on breakthrough infection occurring after vaccination have focused on identifying susceptibility factors such as virus neutralizing titer prior to infection. (*16*) The impact of breakthrough infection on the neutralizing antibody response and how this compares to the response elicited by hybrid immunity remains unclear; we therefore undertook the present study to directly address this gap in knowledge.

## RESULTS

### Cohort and study design

**Fig. 1.**
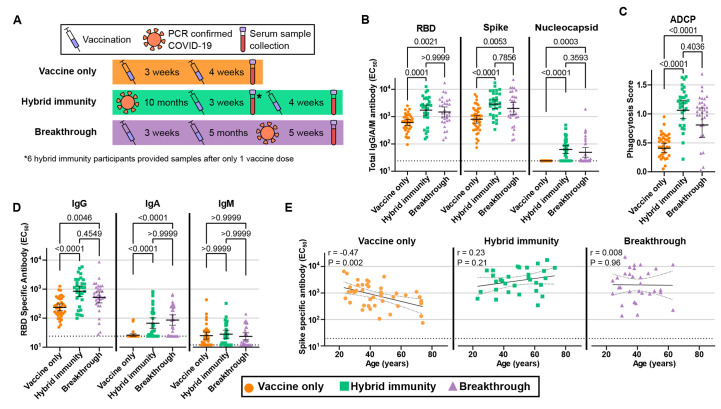
Antibody levels following breakthrough infection, hybrid immunity, and vaccination alone. (A) Schematic depicting the order and approximate time scale of vaccination and natural infection for each group. The blue syringe indicates a dose of vaccine, the orange virus particle indicates PCR confirmed natural infection with SARS-CoV-2, and the purple capped vial indicates serum collection. The asterisk (*) indicates that 6 (out of 31) hybrid immune participants provided serum samples following only a single vaccine dose. (B) IgG/A/M inverse fold-dilution EC_50_ values for sera specific to RBD, full-length spike, and nucleocapsid proteins measured by ELISA. (C) Antibody dependent cellular phagocytosis scores. (D) RBD-specific EC_50_ values for IgG, IgA, and IgM class antibodies measured by ELISA. (E) Correlation between spike-specific EC_50_ values and participant age. Error bars in B and D indicate the geometric mean with the 95% confidence interval, while error bars in C indicate the arithmetic mean with the 95% confidence interval. P values in B-D were calculated with two-tailed Kruskal-Wallis test with Dunn’s multiple comparison correction. Scatter plots in E depict the simple linear fit of age and log transformed EC_50_ values with 95% confidence bands along with the Spearman rank correlation coefficient and two-tailed P value.

We recruited a total of 104 participants (Table 1) consisting of 31 fully vaccinated individuals with PCR-confirmed breakthrough infections, 31 individuals with one (6 individuals) or two vaccine (25 individuals) doses following recovery from COVID-19 (hybrid immunity), and 42 fully vaccinated individuals with no history of COVID-19 or breakthrough infection (Fig. 1A). Ninety-six participants received BNT162b2, 6 received mRNA-1273, and 2 received Ad26.COV2.S. Serum samples were collected from each of the participants, which were then tested for 50% effective antibody concentrations (EC_50_) by enzyme-linked immunosorbent assay (ELISA), and 50% live SARS-CoV-2 neutralizing titer with focus reduction neutralization tests (FRNT_50_) against early lineage strain SARS-CoV-2 (WA1) and clinical isolates of three VOCs: Alpha (B.1.1.7), Beta, (B.1.351), and Delta (B.1.617.2). We performed additional antibody-dependent cellular phagocytosis (ADCP) experiments to evaluate any functional differences in the antibody response of each group.

**Table 1.** Cohort demographics.

**Characteristic**		**Vaccine Only**	**Hybrid Immunity**	**Breakthrough**
		N = 42	N = 31	N = 31
**Sex**				
	Female - N (%)	35 (83.3)	19 (61.3)	24 (77.4)
	Male - N (%)	7 (16.7)	12 (38.7)	7 (22.6)
**Age (yr)**				
	Median [Range]	40 [23-74]	50 [23-73]	38 [24-63]
**Critical time periods (days) - Median [IQR]**				
	Latest vaccine dose to blood draw	24 [17.25-35.75]	25 [17.5-34]	N/A
	PCR positivity to blood draw	N/A	N/A	35 [23-48.5]
	PCR positivity to first vaccine dose	N/A	289 [124-334.5]	N/A
	Second vaccine dose to PCR positive	N/A	N/A	139 [81.5-201.5]
	Days between vaccine doses	21 [21-22]	22 [21-25]	21 [21-23]
**Vaccine type - N (%)**				
	BNT162b2 (Pfizer)	42 (100)	25 (80.6)	29 (93.5)
	mRNA-1273 (Moderna)	0 (0)	5 (16.1)	1 (3.2)
	Ad26.COV2.S (Janssen)	0 (0)	1 (3.2)	1 (3.2)

We first analyzed the hybrid immunity of participants who received only a single vaccine dose compared to those who had received two doses (Fig. S1). All measures of antibody levels, ADCP, and live virus neutralization revealed no significant difference between these two groups. For this reason, we combined these samples into a single group containing participants with both one and two vaccine doses following natural infection, which we henceforth refer to as the hybrid immune group.

### Antibody levels following breakthrough infection, hybrid immunity, and vaccination alone

ELISA geometric mean titers (GMT) EC_50_ values for SARS-CoV-2 spike-specific antibodies were significantly elevated in both the breakthrough (2.5-fold, P = 0.005) and hybrid immune (3.6-fold, P < 0.0001) groups compared to vaccination alone, but we saw no significant difference between the breakthrough and hybrid groups (Fig. 1B). A similar trend was seen for EC_50_ values specific for the spike receptor binding domain (RBD) (Fig. 1B). We additionally confirmed that none of the vaccine-only participants exhibited reactivity against the nucleocapsid (N) protein, supporting lack of previous infection, whereas the breakthrough and hybrid immune groups were 68 and 48 percent N responsive, respectively (Fig. 1B). Opsonization with hybrid immune and breakthrough sera also induced phagocytosis of spike protein-coated particles in an ADCP assay significantly more than vaccination alone, but not compared to each other (Fig. 1C). The levels of IgG and IgA antibodies specific to RBD protein displayed a similar trend to the total EC_50_ levels with significant increases for hybrid immunity and breakthrough compared to vaccination alone, but not compared with each other (Fig. 1D). RBD-specific IgM values were notably low and did not differ significantly between groups. Consistent with previous reports, (*17*) spike-specific antibody levels correlated negatively with age among vaccine-only participants. In contrast, neither the breakthrough nor hybrid immune group recapitulated this correlation, displaying no significant age-related trend (Fig. 1E).

### Neutralizing antibody titers against SARS-CoV-2 and the variants of concern

**Fig. 2.**
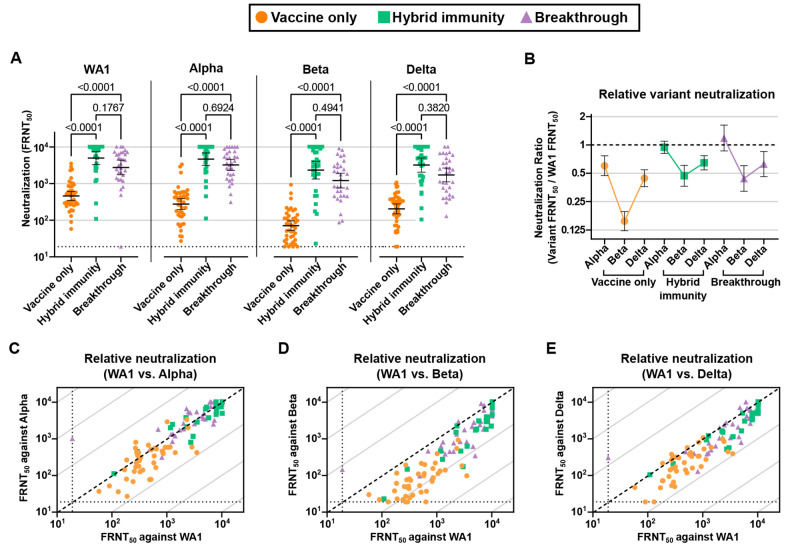
Neutralizing antibody response following breakthrough infection, hybrid immunity, and vaccination alone. (A) Neutralizing antibody titers determined by focus forming assay with clinical isolates of the original strain of SARS-CoV-2 (WA1), Alpha, Beta, and Delta variants. (B) The ratio of Alpha, Beta, and Delta variant neutralization to WA1 neutralization. WA1 neutralizing titer versus Alpha (C), Beta (D), and Delta (E) variant neutralizing titer. The dotted line indicates equal neutralization. Error bars in A and B indicate the geometric mean with the 95% confidence interval. P values in A were two-tailed and calculated with the Kruskal-Wallis method with Dunn’s multiple comparison correction.

We next quantified the functional activity of participants’ immune sera by comparing their neutralization titers against early (WA1) SARS-CoV-2 and selected VOCs. Against all viruses, the trend mirrored that of the antibody EC_50_ levels, with the vaccine-only group FRNT_50_ titers significantly lower than both breakthrough and hybrid immunity, which were comparable with each other (Fig. 2A). The FRNT_50_ GMT of hybrid immune group participants were 10.8, 16.9, 32.8, and 15.7-fold higher than vaccination alone for WA1, Alpha, Beta, and Delta variants, while breakthrough group participants were 6.0, 11.8, 17.0, and 8.5-fold higher than vaccination alone, respectively, all with P < 0.0001. Among vaccine group participants, neutralization of the Beta variant was significantly reduced compared to WA1, while the difference seen for the hybrid immune and breakthrough groups was not significant (Fig. S2).

In addition to eliciting immunity with greater breadth (Fig. 2A), the serum antibody potency across the breadth of VOCs tested was greater for both hybrid immune and breakthrough groups, as measured by an increase in the ratio of variant neutralization over WA1 FRNT50 values against Alpha and Beta for the hybrid immune and breakthrough groups, and against Delta for the hybrid immune group (Fig. 2B and S3). Breakthrough and hybrid immune participants grouped more tightly and displayed variant neutralizing titers closer to that of WA1 (Fig. 2C-E).

### Quality of the neutralizing antibody response

**Fig. 3.**
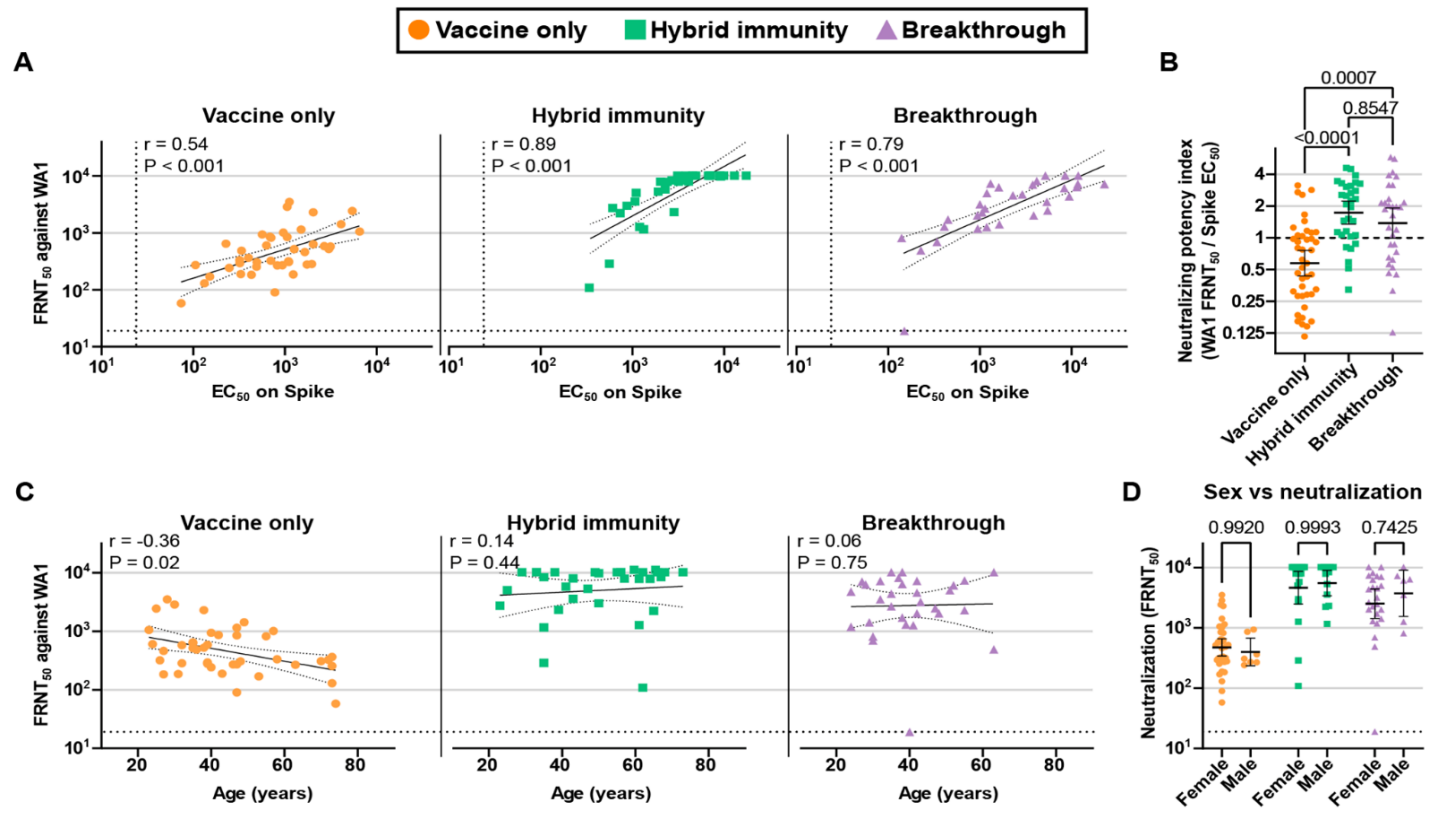
Neutralizing efficiency and correlation with age. (A) Correlation between spike-specific EC_50_ vaules and WA1 neutralizing titers. (B) Serum neutralizing potency index was calculated as the ratio of WA1 neutralizing titer to spike-specific EC_50_ values. (C) Correlation between age and WA1 neutralizing titers. (D) WA1 neutralization by sex. Error bars in B and D indicate the geometric mean with the 95% confidence interval. P values in B were two-tailed and calculated with the Kruskal-Wallis method with Dunn’s multiple comparison correction. P values in D were two-tailed and calculated with using a two-way ANOVA with the Šidák multiple comparison correction. Scatter plots in A depict the simple linear fit of log transformed FRNT_50_ versus log transformed EC_50_ values with 95% confidence bands. Scatter plots in C depict the simple linear fit of log transformed FRNT_50_ versus age with 95% confidence bands. Correlations in A and C show spearman rank correlation coefficients and two-tailed P values.

We also found that hybrid immunity was associated with a remarkable improvement in the proportion of spike-specific antibodies that were also neutralizing. WA1 neutralizing titers correlated with spike-specific antibody levels for all three groups, but the hybrid immune and breakthrough groups correlated more strongly (Fig. 3A). To analyze the efficiency of sera at neutralizing a given virus strain, we determined a neutralizing potency index by calculating the ratio of neutralizing titer (FRNT50) to spike binding EC_50_ values. (*18*) The index expresses a ratio of fold-serum-dilution with 50% neutralization potency to fold-serum-dilution 50% spike binding capacity, or a relative neutralizing antibody to total antibody ratio for a given subject’s serum. The neutralizing potency index was significantly higher among hybrid immune and breakthrough participants than after vaccination alone (Fig. 3B). Lastly, we found that the relationship between age and total antibody levels also extends to neutralizing titer; vaccine-only participants displayed a clear negative correlation with age, while the hybrid immune and breakthrough participants showed no such correlation (Fig. 3C). No association was seen between reported sex and neutralizing titer for any of the groups (Fig. 3D).

## DISCUSSION

Overall, our results show that SARS-CoV-2 infection before or after vaccination gives a significantly larger boost to the neutralizing antibody response compared to two doses of vaccine alone. More importantly, the potency and breadth of the antibody response appears to improve concomitantly. It has been well established that natural infection alone provides short-lived protection from infection, (*17*) showing the importance of vaccination, regardless of infection history. Because vaccination protects against severe disease and death, (*19*) it is safer for individuals to be vaccinated before rather than after natural infection.

The negative correlation between age and neutralizing antibody levels following vaccination alone is an effect that has been previously identified. (*20*) The relationship between age and antibody levels following natural infection is markedly more complex, with a peak in antibody levels seen between the ages of 60 and 80. (*21*) The exact reasons for this association remain to be determined, but one hypothesis is that the greater disease severity among individuals of advanced age leads to an overall greater humoral response. (*18*) These two opposing trends may obscure any age dependence of antibody levels in the present study among patients with humoral responses resulting from both vaccination and natural infection.

Recent studies have suggested that the humoral response continues to develop long after vaccination, with memory B cells at late time points after vaccination showing improved quality and breadth compared to early time points. (*14*, *15*, *22*) Our data cannot separate the contribution of mixed boosting due to the combination of vaccination with natural infection, from the contribution of ongoing memory B cell development during the time between first antigen exposure and most recent boosting, whether from vaccination or breakthrough infection. Future studies with individuals who have been vaccinated and boosted may be able to distinguish between these possibilities, and an early study suggests that booster vaccination 8 months following a second dose leads to improved overall Delta variant neutralizing titers by 6 to 12-fold. (*23*) This appears consistent with the 8.5-fold and 15.7-fold improvements against the Delta variant for the breakthrough and hybrid immune groups, respectively, compared to two vaccine doses alone. This suggests that the magnitude of improvement for booster vaccinations may be similar to those seen with combined vaccination and natural infection, including hybrid immunity with a single dose of mRNA vaccine. This would point to the importance of the memory B cell compartment in generating a robust and variant cross-neutralizing humoral response. While this study focuses on the humoral response, it is known that the cellular response by T cells plays an important role in the responding to SARS-CoV-2 vaccination and infection. (*24*)

COVID-19 vaccines using mRNA technology, including BNT162b2 and mRNA-1273 are the most commonly administered vaccines in the United States, where this study took place, and most of this study’s participants received the BNT162b2 vaccine. However, some participants received the Ad26.COV2.S adenovirus-based vaccine. The majority of hybrid immunity research has focused on mRNA vaccination, but research on adenovirus vaccine hybrid immunity has shown similar improvements to neutralizing titers and variant cross-neutralization. (*25*) While this study was not designed to compare the effectiveness of different vaccination technologies, we do not anticipate any substantial effect due to differences in vaccine types.

Vaccination is highly effective at preventing the most severe outcomes from COVID-19 and should be provided regardless of prior infection status and age. A single dose of vaccine may provide sufficient protection for many individuals with previous SARS-CoV-2 infection. Vaccine availability remains limited in many regions and the shortest path to broad global immunity may be to prioritize administering at least one vaccine dose to as many individuals as possible with a confirmed history of SARS-CoV-2 infection.

## MATERIALS AND METHODS

### Study design

The purpose of this study was to directly compare the humoral immune response among individuals who received COVID-19 vaccines either prior to or following naturally acquired SARS-CoV-2 infection. Serum samples were collected from participants, which were analyzed using enzyme-linked immunosorbent (ELISA) assays, focus reduction neutralization tests, and measurement of antibody dependent cellular phagocytosis. Study participants were selected for inclusion based on a history of both vaccination and previous SARS-CoV-2 infection. Vaccinated controls with no history of previous infection were selected on the basis of sex, age, days between vaccine doses, and the time period since the most recent vaccination.

### Cohort selection and serum collection:

Health care workers at Oregon Health & Science University were recruited and enrolled in the study belonging to three groups: Vaccine-only, hybrid immunity, and breakthrough infection. Written informed consent was obtained at the time of enrollment and study approval was obtained from the OHSU institutional review board (IRB#00022511). Vaccine-only participants were fully vaccinated, defined as having received 2 doses of BNT162b2 or mRNA-1273, or 1 dose of Ad26.COV2.S. Serum samples were collected at least 14 days after the final vaccine dose. Hybrid immune participants had a history of PCR-confirmed diagnosis of COVID-19 at least 10 days prior to vaccination with at least one dose of BNT162b2, mRNA-1273, or Ad26.COV2.S and serum samples were collected at least 10 days after the final vaccine dose. Breakthrough participants were fully vaccinated as defined for the vaccine only group at least 10 days prior to PCR confirmed diagnosis of COVID-19 and serum samples were collected at least 10 days after the date of diagnosis. Sera were obtained by collecting 4-6 mL of whole blood in a BD Vacutainer Plus Plastic Serum Tube, which was centrifuged for 10 min at 1000xg before serum was aliquoted and stored at -20°C. Hybrid immune and breakthrough infection participants were selected based on availability while vaccine-only participants were selected to most closely match the average sex, age, and time since most recent vaccination (or infection for breakthrough) of the other two groups. Participants in these cohorts are previously described. (*20*, *26*)

### Enzyme-linked immunosorbent assays (ELISA):

ELISAs were performed as previously described. (*20*) In 96-well plates (Corning Incorporated, EIA/RIA High binding, Ref #359096). Plates were coated with 100 μL/well of the following proteins at 1 μg/mL in PBS and incubated overnight at 4°C with rocking: SARS-CoV-2 RBD (produced in Expi293F cells and purified using Ni-NTA chromatography), Full-length SARS-CoV-2 spike (Recombinant Spike, SARS-CoV-2 stabilized protein, produced in Expi293F cells, BEI resources #NR-52724), Nucleocapsid (SARS-CoV-2 Nucleocapsid-His, insect cell-expressed, SinoBio Cat: 40588-V08B, Item #NR-53797, lot #MF14DE1611). Plates were washed three times with 0.05% v/v Tween-20 in PBS (wash buffer) and blocked with 150 μL/well 5% nonfat dry milk powder in wash buffer (blocking buffer) at room temperature of approximately 20°C (RT) for 1 hour with rocking. Breakthrough and control sera were aliquoted and frozen in dilution plates then resuspended in blocking buffer; sera were diluted and added to ELISA plates 100 μL/well (6 × 4-fold dilutions from 1:50 to 1:51,200), except for IgM (6 × 3-fold dilutions from 1:25 to 1:6075). Sera was incubated for 1 hour at RT before plates were filled three times with wash buffer. Secondary antibodies were added to plates at 100 μL/well depending on the intended readout: Goat anti-human IGG/A/M-HRP at 1:10,000 (Invitrogen, Ref #A18847), anti-human IgA-HRP at 1:3,000 (BioLegend, Ref #411002), Mouse anti-human IgG-HRP Clone G18-145 at 1:3,000 (BD Biosciences, Ref #555788), Goat anti-human IgM-HRP at 1:3,000 (Bethyl Laboratories, Ref #A80-100P). Plates were incubated protected from light with secondary at RT for 1 hour with rocking, then filled three times with wash buffer prior to the development with o-phenylenediamine dihydrochloride (OPD, Thermo Scientific #34005) according to the manufacturer’s instructions. The reaction was stopped after 25 min using an equivalent volume of 1 M HCl; optical density was measured at 492 nm using a CLARIOstar plate reader. Normalized A_492_ values were calculated by subtracting the average of negative control wells and dividing by the 99^th^ percentile of all wells from the same experiment. A dilution series of positive control serum was included on each plate to verify appropriate performance of the assay.

### Cell culture:

Vero E6 monkey kidney epithelial cells (CRL-1586) were obtained from ATCC and maintained in tissue culture-treated vessels in Dulbecco's Modified Eagle Medium (DMEM), 10% fetal bovine serum (FBS), 1% nonessential amino acids (NEAA), 1% penicillin-streptomycin (PS) (complete media) in tissue culture conditions (TCC) of 100% relative humidity, 37°C, and 5% CO_2_. THP-1 (ATCC, TIB-202) human monocyte cells were obtained from ATCC and maintained in suspension culture in tissue culture treated vessels in Roswell Park Memorial Institute medium (RPMI-1640) supplemented with 10% FBS, 1% NEAA, and 1% PS (THP-1 media).

### SARS-CoV-2 growth and titration:

SARS-CoV-2 isolates USA-WA1/2020 [lineage A] (NR-52281), USA/CA_CDC_5574/2020 [lineage B.1.1.7 – alpha] (NR-54011), hCoV-19/South Africa/KRISP-K005325/2020 [lineage B.1.351 – beta] (NR-54009), hCoV-19/USA/PHC658/2021 [lineage B.1.617.2 – delta] (NR-55611) were obtained from BEI Resources. Viral stocks were propagated as previously described. (*5*) Sub-confluent Vero E6 cells were infected at an MOI of 0.05 in a minimal volume (0.01 mL/cm2) of Opti-MEM + 2% FBS (dilution media) for 1 hour at TCC then 0.1 mL/cm2 additional complete media was added and incubated for 24 hours at TCC. Culture supernatant was centrifuged for 10 min at 1000xg and frozen at -80°C in aliquots. Titration was performed on clear 96 well tissue culture plates containing 70–90% confluent (at the time of infection) Vero E6 cells. 8 × 10-fold dilutions were prepared in dilution media and 30 μL/well of diluted virus was incubated with the cells for 1 hour at TCC before further addition of Opti-MEM, 2% FBS, 1% methylcellulose (overlay media) and incubation for 24 hours at TCC. Plates were then fixed by soaking in 4% formaldehyde in PBS for 1 hour then removing from BSL-3 following institutional biosafety protocols. Cells were permeabilized in 0.1% bovine serum albumin and 0.1% saponin in PBS (perm buffer) for 30 min, then with polyclonal anti-SARS-CoV-2 alpaca serum (Capralogics Inc.) (1:5000 in perm buffer) overnight at 4°C. Plates were washed three times with 0.01% Tween-20 in PBS (focus wash buffer), then incubated for 2 hours at RT with 1:20,000 anti-alpaca-HRP (Novus #NB7242). Plates were filled three times with focus wash buffer, then incubated with TrueBlue (Sera Care #5510-0030) for 30 min or until sufficiently developed for imaging. Well images were captures with a CTL Immunospot Analyzer and counted with Viridot (1.0) in R (3.6.3). (*27*) Viral stock titers in focus forming units (FFU) were calculated from the dilution factor and volume used during infection.

### Focus reduction neutralization test (FRNT):

FRNT assays were carried out as described. (*5*) Duplicate 5x4.7-fold (1:10-1:4879) serial dilutions of participant sera were prepared in 96-well plates. An equal volume of dilution media containing approximately 50 FFU of SARS-CoV-2 or variant was added to each well (final dilutions of sera, 1:20 – 1:9760) and incubated 1 hour at TCC. Virus-serum mixtures were used to infect Vero E6 cells in 96-well plates as described above in the titration assay. Each plate contained 16 virus-only control wells, one for each serum dilution series. Fixation, development, and counting of FRNT plates was carried out as described above in the titration assay. Percent neutralization values were calculated for each well as the focus count divided by the average focus count of virus-only control wells from the same plate.

### Antibody Dependent Cellular Phagocytosis (ADCP):

ADCP assay was adapted from a protocol described previously. (*28*) Biotinylated RBD incubated at 1μg/ml with fluorescent neutravidin beads (Invitrogen, F8775) for 2 hours at RT; beads were washed twice with 1% BSA in PBS (dilution buffer) and resuspended at a final dilution of 1:100 in dilution buffer. In a 96-well plate, 10μL of resuspended bead solution was incubated with 10μL of diluted serum from study subjects for 2 hours at 37°C. After serum pre-treatment, 2x10^4^ THP-1 cells were added to each well in 80μL THP-1 media and incubated overnight in TCC. The following morning, 100 μL of 4% paraformaldehyde was added to each well and incubated at least 30 min at RT before analysis on a CytoFLEX flow cytometer (Beckman Coulter). Samples were mixed for 3 s prior to analysis and samples were injected until at least 2500 cell events were recorded per sample. Phagocytosis scores are reported as the product of percent bead-positive cells and mean fluorescence intensity of bead-positive cells, then divided by 10^6^ for presentation. Three replicate experiments were performed for each participant serum sample, the average of which was used for further analysis. The gating strategy with representative data are presented in Fig. S4.

### Statistical analysis:

FRNT_50_ and EC_50_ values were calculated by fitting percent neutralization or normalized A_492_ values to a dose-response curve as previously described. (*5*) Final FRNT_50_ values below the limit of detection (1:20) were set to 1:19. Final EC_50_ values below the limit of detection of 1:25 for N, Spike, RBD, IgG, IgA were set to 1:24 and values below 1:12.5 for IgM was set to 1:12. Aggregated EC_50_ and FRNT_50_ values were analyzed and plotted in Graphpad Prism (9.2.0). Dot plots of EC_50_ and FRNT_50_ values were generated on a log transformed axis with error bars showing the geometric mean and 95% confidence interval. Phagocytosis score and Neutralization ratio were plotted on a linear axis with error bars showing the arithmetic mean and 95% confidence interval. P values for dot plots were two-tailed and calculated using the Kruskal-Wallis test with Dunn’s multiple comparison correction. P values for reported sex versus neutralization were two-tailed and calculated by group using a two-way ANOVA with the Šidák multiple comparison correction. Scatter plots were prepared by first log transforming FRNT_50_ and EC_50_ data then performing simple linear fitting and plotting the 95% confidence bands. Correlations were calculated using Spearman’s correlation and two-tailed P values were calculated for the 95% confidence interval.

## Supplementary Materials

### Other Supplementary Material for this manuscript includes the following:

MDAR Reproducibility Checklist
