# Supplementary material for: Vaccination before or after SARS-CoV-2 infection leads to robust humoral response and antibodies that effectively neutralize variants
Source: Sci Immunol. 2022 Jan 25:eabn8014. doi: 10.1126/sciimmunol.abn8014 (PMC8939472; doi:10.1126/sciimmunol.abn8014)
Supplement: Supplementary file 1 — Figs. S1 to S4 [file sciimmunol.abn8014_sm.pdf]

Science Immunology
AAAS

Supplementary Materials for

Vaccination before or after SARS-CoV-2 infection leads to robust humoral response and antibodies that effectively neutralize variants

Timothy A. Bates *et al.*

Corresponding authors: William B. Messer, messer@ohsu.edu; Marcel E. Curlin, curlin@ohsu.edu;
Fikadu G. Tafesse, tafesse@ohsu.edu

DOI: 10.1126/sciimmunol.abn8014

The PDF file includes:

Figs. S1 to S4

Other Supplementary Material for this manuscript includes the following:

MDAR Reproducibility Checklist

Supplementary Materials

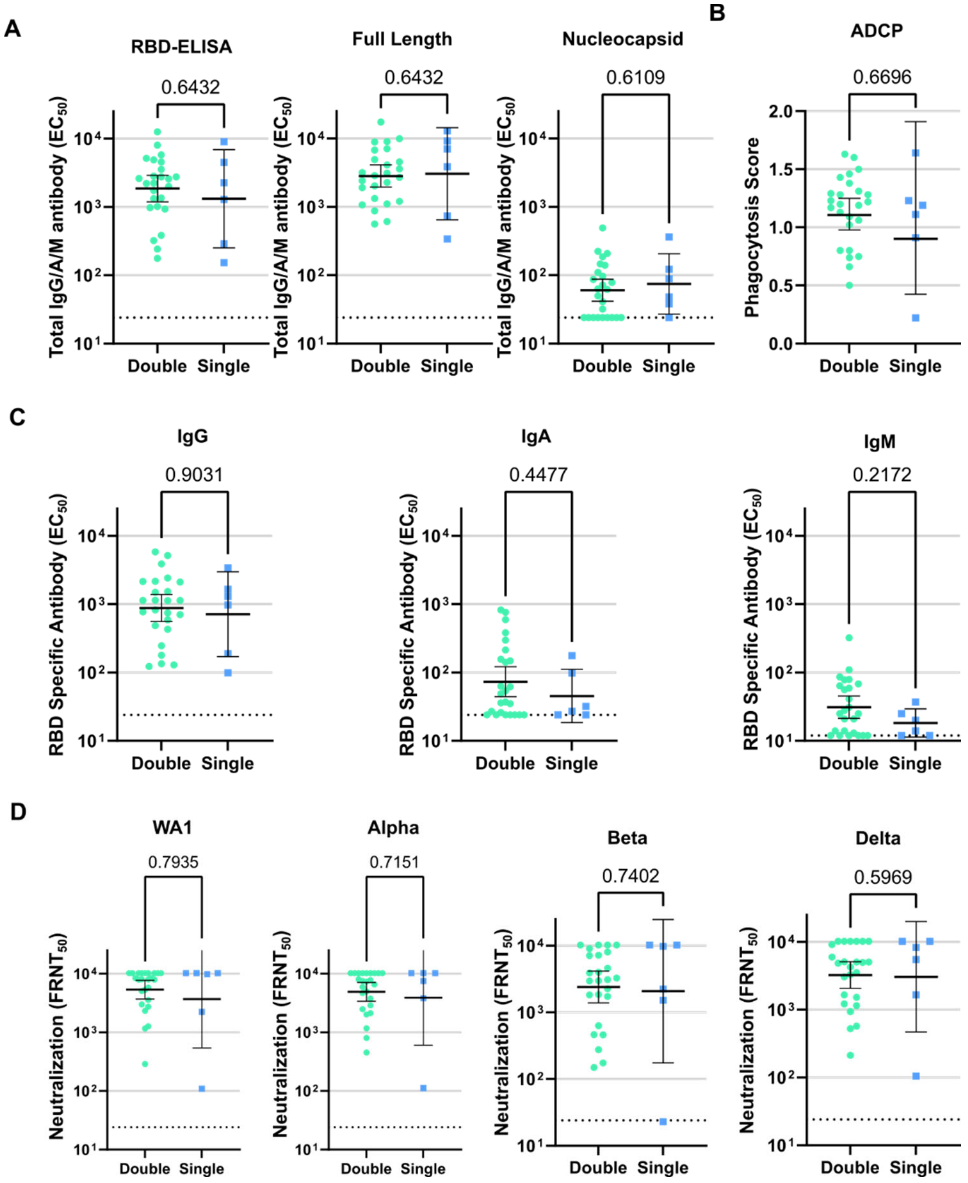

Supplemental Figure 1: One versus two vaccine dose hybrid immunity. (A) Total IgG/A/M antibody levels determined by ELISA. (B) Antibody dependent phagocytosis scores. (C) RBD-specific antibody levels by class. (D) Live virus neutralization by variant. Error bars show the geometric mean (arithmetic mean for ADCP) with 95% confidence intervals. P values are two-tailed and were calculated using the Kruskal-Wallis test.

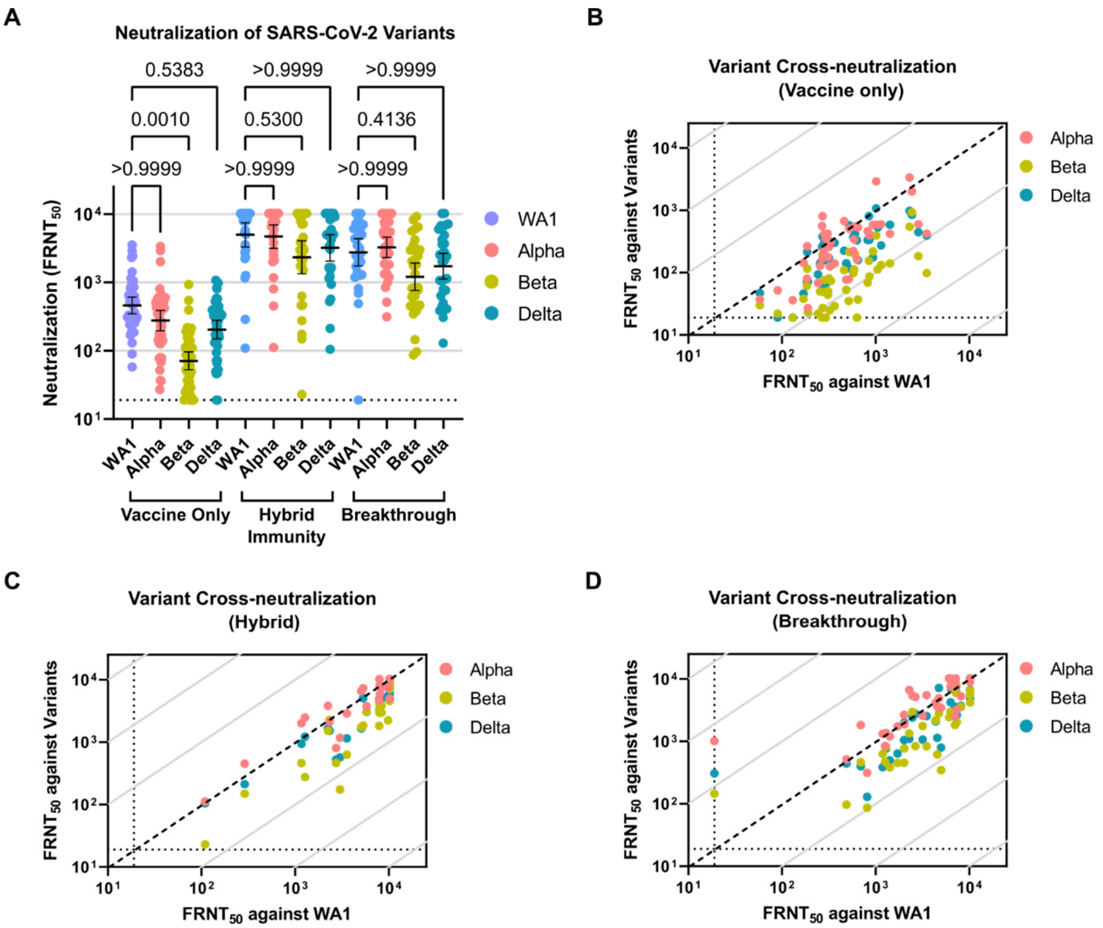

Supplemental Figure 2: Neutralization by variant. (A) Neutralization of live SARS-CoV-2 clinical isolates organized by group. WA1 neutralizing titer versus each variant for vaccine only (B), hybrid immunity (C), and breakthrough (D) groups. The dotted line indicates equal neutralization of variants and WA1 for each participant.

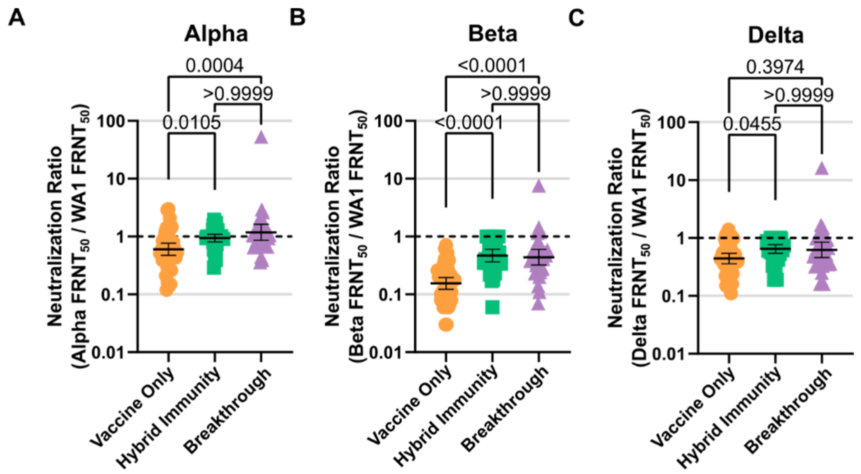

Supplemental Figure 3: Variant neutralization ratios. Ratio of variant neutralization over WA1 neutralization titers for Alpha (A), Beta (B), and Delta (C). The dotted lines indicate equal neutralization of variant and WA1. Error bars indicate the geometric mean and 95% confidence interval. P values are two-tailed and were calculated with the Kruskal-Wallis method with Dunn’s multiple comparison correction.

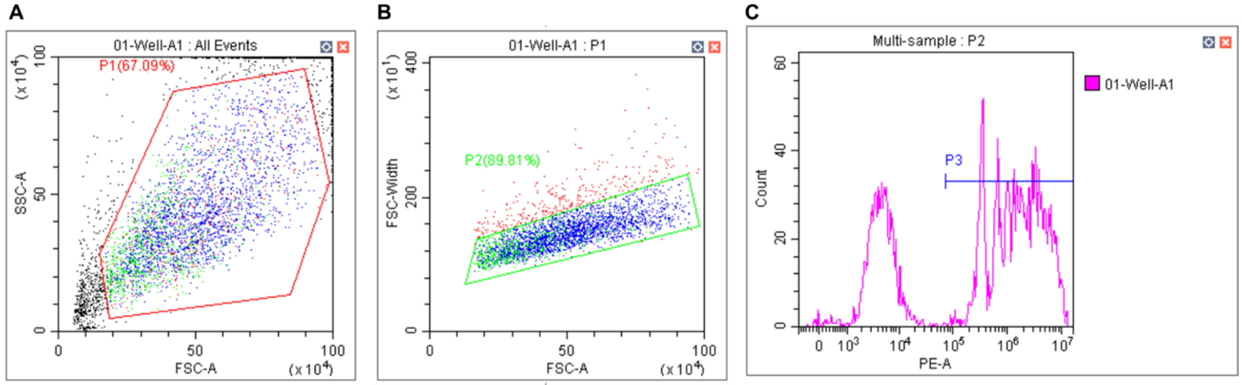

Supplemental Figure 4: ADCP flow gating scheme. (A) Intact cells were isolated from free beads and cell debris based on forward and side scatter. (B) Singlet cells were isolated from doublets/aggregates by forward scatter width. (C) Group P3 indicates cells which have phagocytosed at least one fluorescent bead, whereas group P2 represents all single cells including those which have not taken up any beads.
